# Optical Imaging, Optical Sensing and Devices

**DOI:** 10.3390/s23062882

**Published:** 2023-03-07

**Authors:** Wen Chen, Ming Tang, Liang Wang

**Affiliations:** 1 Department of Electronic and Information Engineering & Photonics Research Institute, The Hong Kong Polytechnic University, Hong Kong, China; 2 National Engineering Laboratory for Next Generation Internet Access System, School of Optics and Electronic Information & Wuhan National Lab for Optoelectronics (WNLO), Huazhong University of Science and Technology, Wuhan 430074, China

Technological advances have recently provided an excellent opportunity for development in optical fields, e.g., optical imaging, sensing and devices. The developments of optical imaging and sensing are also related to optical devices, e.g., laser and image sensors. Laser technologies and image sensors play an important role in various applications, e.g., optical imaging, optical security, remote sensing and three-dimensional (3D) reconstruction. A considerable amount of thecurrent research is dedicated to solving the problems encountered in optical imaging and sensing.

This Special Issue contains the latest research advances in optical imaging, optical sensing and optical devices, emphasizing the integration of opto-electric measurement and computational methods, theoretical to experimental demonstration, and related applications. This Special Issue aims to compile contributions by outstanding international leaders, researchers, scientists and engineers from various interdisciplinary fields to present their work in optical imaging, sensing and devices. This Special Issue also focuses on the current state-of-the-art of optical imaging, sensing and devices, covering recent developments in new imaging and sensing systems and emerging applications.

Kim et al. [1] report a simultaneous frequency stabilization of two 780-nm external cavity diode lasers using a precision wavelength meter. It is shown that the laser stabilization technique can operate at a broad wavelength range without a radio frequency element, which could be utilized for most of the single-mode lasers operating from near-UV to the telecom band. Huang et al. [2] review a laser named the Fourier domain mode-locked (FDML) laser, which has ahigher sweep rate and alarger sweep range over conventional short-cavity lasers. FDML was proposed to overcome the limitations of buildup time by inserting a long fiber delay in the cavity to store the whole swept signal.

Skvortsov et al. [3] study the surface-enhanced Raman spectra of amino acids D-alanine and DL-serine and their mixture on silver nanoisland films immersed in phosphate-buffered saline solution at millimolar amino acid concentrations. The surface-enhanced Raman spectra have a broad and bright future for optical imaging. Zheng et al. [4] study the beam homogenization system of a semiconductor laser based on a homogenizing pipe, aiming at applying laser active imaging detection. Furthermore, research results have particular reference values for other applications requiring a uniform laser spot, such as medical treatment and welding. Zhang et al. [5] propose a polarization phasor imaging method for image recovery in foggy scenes with the assistance of ToF cameras. With the introduction of metalens, Qu et al. [6] propose a dual-wavelength achromatic metalens that generates one or two foci according to the polarization of the incident. The proposed method is demonstrated to open the path for a combination of multi-wavelength imaging and chiral imaging, which may find potential applications, such as achromatic optical devices and polarization-controlled biomedical molecular imaging systems. In addition to two-dimensional optical imaging, Feng et al. [7] propose a 3D reconstruction method based on phase similarity, which can increase the accuracy of depth estimation and the scope of applicability of an epipolar plane image (EPI). Compared with traditional EPI, their method can make EPI perform well in a single scene or blurred texture situations and maintain high accuracy. Li et al. [8] propose a new multi-image encryption method based on sinusoidal stripe coding frequency multiplexing, and deep learning is applied to realize the encryption of a greater number of images. The efficiency of the proposed encryption method is verified in terms of a histogram, adjacent pixels correlation, anti-noise attack and resistance to occlusion attacks.

Four research articles in this Special Issue focus on optical sensing and devices. Recently, optical sensors attracted attention because of their high sensitivity, compact size, anti-electromagnetic interference and low cost. Li et al. [9] propose a large-scale shaft diameter precision measurement method based on a dual camera system to balance the accuracy and measurement range. Feng et al. [10] propose a novel temperature-compensated multi-point strain sensing system based on cascaded fiber Bragg grating (FBG) and optical frequency-modulated continuous wave (FMCW) interferometry. It is demonstrated that the sensing system using optical FMCW interferometry combined with cascaded FBGs successfully monitors axial strain distribution and also realizes the function of temperature compensation. The system has practical significance in the field of quasi-distributed strain measurement. Yu et al. [11] propose a few-mode fiber (FMF) characterization system based on the spatial and spectral imaging technique. The proposed system spectrally characterizes few-mode fiber by resolving interference information from the superimposed optical field and has a simple structure and easy operation, providing a guide for the FMF design and the FMF experimental optimization. Li et al. [12] study transmission modes’ field distribution and effective refractive index in single-core six-hole optical fiber. Compared to other strain sensors, the designed sensor has good properties, e.g., low cost, small size and high sensitivity.

In conclusion, this Special Issue presents new developments inoptical imaging, sensing and devices, i.e., laser technologies, high-quality optical imaging, 3D imaging, phase imaging, optical encryption and optical sensors. Hopefully, the topics investigated in this Special Issue could support optical imaging and optical sensing with further device advancements.
